# Positive ecological effects of wind farms on vegetation in China’s Gobi desert

**DOI:** 10.1038/s41598-019-42569-0

**Published:** 2019-04-26

**Authors:** Kang Xu, Lingchao He, Hanjian Hu, Shun Liu, Yuanyuan Du, Zhiwei Wang, Yan Li, Liyan Li, Alamgir Khan, Genxuan Wang

**Affiliations:** College of Life Sciences, Zhejiang University, 866 Yuhangtang Road, Hangzhou, 310058 P. R. China

**Keywords:** Ecology, Environmental impact

## Abstract

With the rapid development of wind power, there are increasing concerns about the negative ecological effects of its construction and operation. However, previous studies have mainly focused on the effects of wind farms on flying fauna (i.e., birds and bats) or climate change separately from communities or ecosystems, and little attention has been paid to vegetation during wind farm operation. Furthermore, few studies have referred to vulnerable ecosystems with low biomass and biodiversity. In this research, a field study was conducted to investigate the effects of wind farms on the individual traits, community structures and ecosystem functions of Gobi Desert ecosystems. The effects were measured by comparing interfering areas (IAs, located between 40 m and 90 m in the downstream direction of the wind turbine) with non-interfering areas (NIAs, located over 200 m from the wind turbine matrixes). The results showed that (1) plant individuals in IAs were less stressed and in better physiological states than those in NIAs; (2) for community structures, IA plants tended to be shorter and denser and had a higher coverage condition than that of NIA plants; and (3) ecosystem functions in IAs were significantly improved due to the existence of shrubs and higher biomass. Meanwhile, significant correlations were identified between the wind wake caused by the large spinning blades and the community structures. Constructing wind turbines in the Gobi Desert is a win-win strategy that both contributes to the growth of desert vegetation with a favourable microclimate and sufficiently utilizes wind power to produce clean energy.

## Introduction

Compared to traditional fossil fuels, such as petroleum, coal and natural gas, wind energy is an efficient renewable energy source that can significantly alleviate severe energy shortages, air pollution, and environmental degradation^1^. Hence, under the current energy policies that encourage low or zero greenhouse gas emissions, wind power has become one of the fastest-growing energy sources, and the global cumulative installed wind capacity nearly doubled from 283 GW in 2012 to 539 GW in 2017^2^. Wind energy supplied 3.7% of the world’s electricity demand in 2015. It is expected to reach 15–18% by 2050 at a capacity of 2.3–2.8 TW, which represents a shift from fossil fuels to renewable energy resources^1,3^.

The number of wind farms has been increasing rapidly in response to the demand for renewable energy. In contrast to the beginning period of the 1980s, when wind energy was thought to be absolutely “clean” and completely free from having any risk of environmental impact, the rapid development and spatial extent scale of wind farm facilities has raised concerns about their negative effects^4^. First, the construction of wind farms may result in an increasing risk of collision and habitat loss of flying fauna (especially birds and bats)^5–8^. Second, the large number of wind farms may produce noticeable climatic change due to the alteration of kinetic energy fluxes and the encompassed turbulent flow phenomena^9^. Previous research that focused on global climate change documented that a net near-surface warming of 0.7–1.5 °C, together with zonal precipitation that decreased by approximately 1%, occurred in areas covered by wind farms according both to satellite observations^9–11^ and model simulations^12–14^. However, the effects of wind farms on vegetation have not been explored^3^. For example, life cycle assessments, which have been widely used to quantify the environmental impacts of wind farms across the life cycle^15–18^, have often excluded the impact on vegetation, even though there has been recent evidence of change^19^.

China has led the global wind market for nine consecutive years, i.e., since 2009, accounting for 35% of the global installed capacity^2^. However, the development pattern of China’s wind power is different from that of traditional wind energy powerhouses. In most European countries, wind farms are mainly located in areas with high biodiversity, such as in areas along coastlines, while inland wind farms rather than coastal and offshore wind farms dominate in China^20^. Over one-third of the national installed wind capacity is located in the sparsely populated drylands or in the Gobi Deserts in north-western China; these areas are rich in wind resources and are suitable for massive wind power development^21^. Guazhou, which is in Gansu Province and is known as the “World Wind Library”^22^, owns the largest wind farm cohort in the world. Currently, the wind energy generated in Guazhou can reach levels greater than 20 GW, which is roughly equivalent to that of all of Spain (i.e., 23 GW in 2017, ranked fifth in the world after Germany)^2,23^. Furthermore, we should not overlook the important roles of the desert ecosystems in terms of biodiversity and carbon sinking; additionally, deserts are home to 6% of the world’s population, and together with other dryland ecosystems, harbour almost one-third of the terrestrial global carbon stock^24^. However, current research on the ecological effects and the evaluation of wind development in deserts has not kept pace with recent progress and is largely dwarfed by the large size of wind farms in these areas.

This study explores the effects of wind farms on vegetation by using the Gobi wind farms in Guazhou as a case study. The differences in the spatial patterns around the wind turbines in terms of individual traits (i.e., metabolic scaling exponent and metabolic level), community structures (i.e., vegetation coverage, community height, and density), and ecosystem functions (aboveground biomass, AGB) as well as their response to the wind wake were investigated in the Gobi Desert in Guazhou. The objectives of this study are to (1) explore whether inland wind farms in deserts have significant effects on vegetation and whether the effects are positive or negative; (2) quantify the vegetation variations caused by the operation of wind farms in ecosystems; and (3) analyse whether the effects are related to local climate change (i.e., the change in wind velocity).

## Materials and Methods

### Study area

The field study was carried out at the First Guazhou Wind Farm (95°17′12”−95°34′43″E, 40°45′31–40°36′2″N), Guazhou City, Gansu Province, which is in the arid area of north-western China. Guazhou experiences a typical continental climate, with an uneven precipitation and temperature pattern. The mean annual precipitation is 52.6 mm, with over 58.1% occurring from June to August, the mean annual temperature is 9.6 °C, and the highest temperature occurs in July and August. The prevailing wind is in the east direction. Winds predominantly (80%) flowed from the east in the year prior to our field study (from June 2015 to May 2016), which is in accordance with the prevailing wind direction. All climatic data are from the China Meteorological Data Sharing Service Center (2007–2016, http://data.cma.cn). The First Guazhou Wind Farm is located 7 km north of the county centre in the Gobi Desert; construction began in 2009, and the wind farm began operating in 2010. No vegetation damage other than infrastructure occupation occurred during the construction period, and no man-made maintenance of the ecosystem was carried out during the operation period. Because it has been in operation for nearly one decade, the ecosystem has adapted to the existence of wind turbines and become stable. The design of the wind turbine generator system is based on FD77-1500, which features a rotor diameter of 77 m and a hub height of 82 m. The foundation is a shallow buried reinforced concrete bucket foundation, which is 16 m in diameter, 3 m in depth and concreted *in situ*; after excavation, the hole was filled with backfill soil (631 m^3^), concrete (332.5 m^3^) and reinforced steel (29.5 t). The study site is a typical shrub-dominated desert that has formed a stable self-organized patchiness pattern^25^.

### Field study design

**Figure 1:**
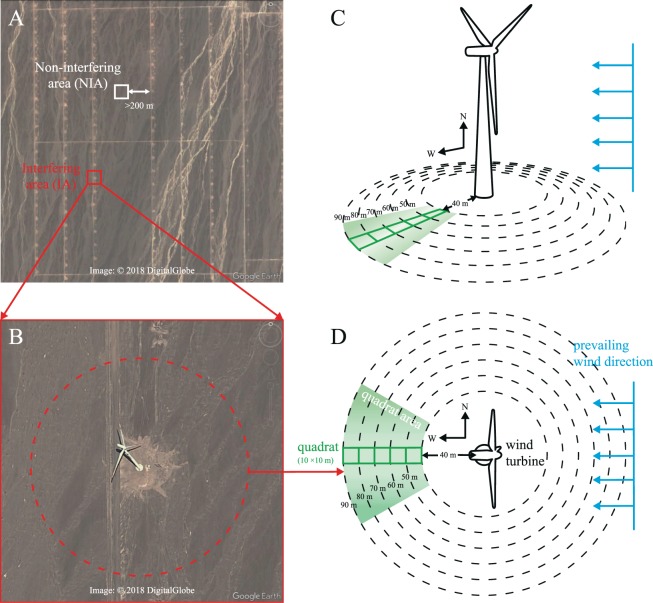
The illustration of the field study design. Matrixes of wind turbines in the wind farm (**A**) and a satellite picture of a single wind turbine (**B**). White and red squares indicate the different experimental areas. Images (**A**,**B**) are from Google Earth (version 7.1.5.1557). The pattern of the experimental design is shown in the horizontal view (**C)** and in the vertical view (**D**). Dashed circles and ellipses divide the five experimental annuli along the radius to the centre of a wind turbine. Blue arrows show the wind direction during the experiment. Each of the green square represents a 10 × 10 m quadrat, and the light green sector is the area used to establish quadrats.

The experiment was conducted between June and August 2016. Seventy-five interfering area (IA) quadrats were established in the downstream direction (west) of 15 independent wind turbines. The IA quadrats were distributed along a distance gradient of 40 m to 90 m from the centre of a wind turbine (Fig. 1C,D) because each generator requires an area of clear ground with a radius of 30–40 m, and this area represents the transition from the foundation to natural vegetation areas and must be excluded. The identification of the farthest distance was based on the stable wind velocity according to the extended Jensen model^26,27^, and previous studies have shown that the effect on birds is not significant at distances exceeding 100 m^7,28–30^. A total of 30 non-interfering area (NIA) quadrats were randomly established away from the wind turbines to minimize the spatial heterogeneity (>200 m between wind turbine matrixes, Fig. 1A). The threshold of 200 m was based on the distance between single wind turbines because the design of the wind farm layout must maximize the efficiency and minimize the power loss caused by wind wakes^27^. All sampling quadrats were 10 × 10 m in size and were selected at sites that were far from transmission lines, roads (Fig. 1B) and ditches (Fig. 1A) to reduce possible disturbances.

### Sampling and measurements

Due to the harsh environment, only three species grow in the study site, including *Bassia dasyphylla* (Fisch. et Mey.) O. Kuntze, *Nitraria sphaerocarpa* Maxim. and *Ephedra przewalskii* Stapf. The first species is an annual grass, and the latter two species are perennial shrubs. Three individual species were sampled in all quadrats. We measured the crown radius (crowns were viewed as ellipses in a two-dimensional space, and the geometric mean of its two axes was used as an estimation of the radius) and the height of each plant. The density was determined by dividing the number of plants in each quadrat and the area of the quadrat. The 5TE soil moisture, temperature and electrical conductivity sensor was used to measure the volumetric water content and the soil temperature. Soil moisture and temperature were continuously measured in each quadrat during the sampling. The sensor was buried at a depth of 30 centimetres during measuring. To minimize damage, only a tiny fraction of a whole plant was clipped, and the total AGB and leaf mass were calculated based on the proportion of the horizontal section area of the clipped parts over that of the whole plant based on the horizontally captured images. The leaves were separated from the stems to weigh the respective masses, and the green photosynthetic shoots of *Nitraria sphaerocarpa* Maxim. were classified as leaf parts, while the yellow shoots were treated as stem parts. Dry mass was weighed after oven drying at 70 °C for 48 h in the laboratory^31^.

### Calculations

#### Metabolic scaling model

The metabolic theory of ecology (MTE) describes the allometric scaling relation of metabolic rate and body size. Its central equation is based on a power law^32,33^, which can be expressed as follows:

1 $$Q={Q}_{0}{M}^{\alpha }$$

where *Q* is the mass-specific metabolic rate (i.e., respiration rate or photosynthetic rate), *Q*_0_ is a normalization constant and can be treated as an indicator of metabolic level in Glazier’s metabolic-level boundary hypothesis^34^. *M* is the body size, and *α* is the metabolic scaling exponent, which has been shown to vary both among taxa^35^ and among physiological states^36^.

In theory, the net photosynthetic rate has been shown to be isometric with respect to the total photosynthetic leaf mass (*M*_*l*_)^37^. Therefore, we used the allometric relation of leaf mass and AGB

2 $${M}_{l}={Q}_{0}{{\rm{AGB}}}^{\alpha }$$

to describe the metabolic condition of an individual based on the variation in biomass partitioning.

#### Wind wake model

We used the well-known extended Jensen model to simulate the wake of wind velocity around a wind turbine at hub height^26,27,38^. The core of this model considers the wind turbine as having a higher surface roughness. The ratio of the wake downflow *v*(*x*) (at the downstream distance *x* from the wind turbine) over the inflow velocity *v*_0_ can be expressed as follows:

3 $$\{\begin{array}{c}\frac{v(x)}{{v}_{0}}=1-a(1+\frac{\frac{x}{d}}{\sqrt{{(\frac{x}{d})}^{2}+\frac{1}{4}}}),x\le {x}_{0}\\ \frac{v(x)}{{v}_{0}}=(1-exp(-\,\beta \frac{x}{d})[1-(1-\sqrt{1-{C}_{T}}){(\frac{d}{d+2kx})}^{2}],x > {x}_{0}\end{array}$$

where *a* is the flow induction factor, which is defined as *u* = (1-*a*)*v*_0_ (*u* is the wind velocity in the hub plane) according to actuator disc theory; *d* is the turbine diameter; *C*_*T*_ is the thrust coefficient t derived from a: *C*_*T*_ = 4*a*(1 − *a*); and *k* is the wake expansion coefficient. In this study, *C*_*T*_ = 0.743, *a* = 0.2465, *d* = 77 m, *β* = 1, and *k* = 0.0939 according to the manual of wind turbine generator system: FD77-1500 and the local wind velocity.

Because the wake is also embedded in the atmospheric boundary layer (the bottom part of the atmosphere, which is directly influenced by its contact with the planetary surface) with its mean velocity shear, we used a popular power law derived from a large cohort of empirical data to evaluate the change in the wind velocity in the vertical profile as follows:

4 $$\frac{v({h}_{1})}{v({h}_{2})}={(\frac{{h}_{1}}{{h}_{2}})}^{\alpha }$$

where *v*(*h*_1_) and *v*(*h*_2_) are the wind velocities at heights *h*_1_ and *h*_2_, respectively, and *α* is the wind shear exponent. Here, we used the one-seventh power law, where *α* = 0.14, as many investigators have done for fairly flat terrains^39^.

### Statistical analysis

The regressions, ANOVAs and correlation analysis were conducted in **R** (3.1.3). Ordinary least square regression analyses were used to establish scaling relationships between leaf mass and aboveground biomass. One-way ANOVAs were performed to test the differences between normally distributed groups. To test the differences between different groups of non-normal data, Kruskal-Wallis (KW) post hoc tests were employed using the *pgirmess*^40^ library in **R** (3.1.3). To assess how the wind effects changed the relation between each pair of functional traits, classical Pearson’s correlation tests were performed to verify the relationships of vegetation traits within IA and NIA plots.

## Results

### Individual physiological states

**Figure 2:**
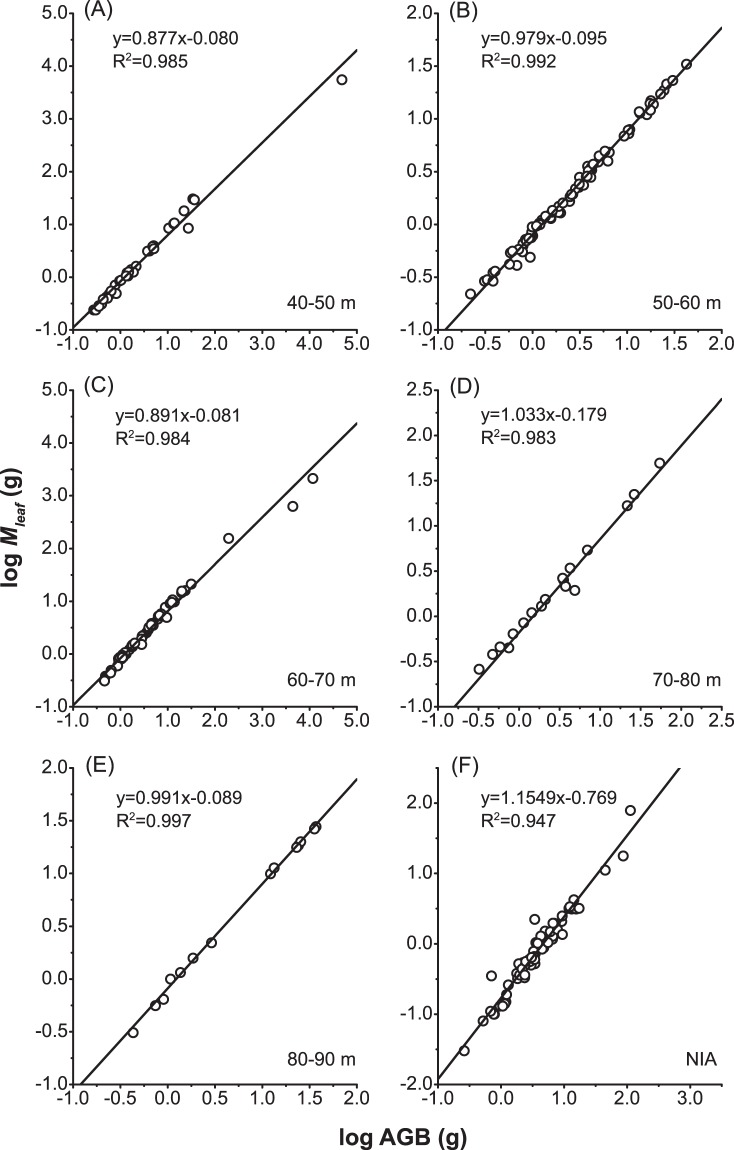
Scaling relationships between leaf mass (*M*_*leaf*_) and aboveground biomass (AGB) of plant individuals at different distances from the wind turbines. The distances in (**A**) to (**E**) are 40–50, 50–60, 60–70, 70–80, 80–90 m, respectively, and (**F**) represents individuals in non-interfering areas (NIAs).

**Figure 3:**
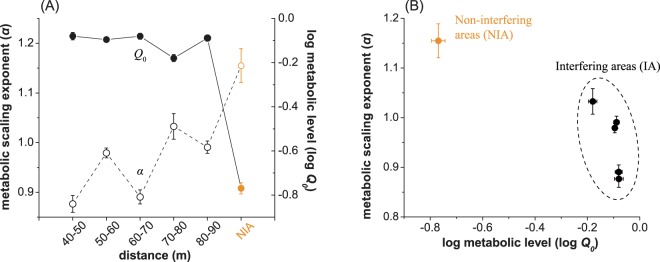
The distributions of the metabolic scaling exponent and metabolic level of non-interfering and interfering plant individuals. (**A**) Metabolic scaling exponent (*α*) and log metabolic level (*Q*_0_) at different distances around the wind turbines. Empty circles denote α, and solid circles indicate *Q*_0_. (**B**) Metabolic scaling exponent versus log metabolic level (*Q*_0_). Orange and black indicate the non-interfering area (NIA) and interfering area (IA) individuals, respectively, in both (**A**,**B**). Error bars indicate standard errors.

Regression analyses of the log10-transformed data revealed linear relationships between the leaf biomass and the AGB of individuals (Table 1, Fig. 2). In particular, with *P* < 0.001 and R^2^ > 0.983, the slopes (i.e., the metabolic scaling exponents) at distances from 40 m to 90 m were 0.877, 0.979, 0.891, 1.033 and 0.991, respectively (Fig. 2). These values were statistically higher than the value of 0.75 (*P* < 0.001), i.e., the universal scaling exponent in the MTE, but lower than 1.15 (*P* < 0.001) for the NIA plants (Fig. 2A–E). Compared with the NIA plots, the metabolic level (*Q*_0_) was significantly higher in the IA plots, ranging from −0.18 to −0.08, where the lower end corresponds to a distance of 70–80 m (*P* < 0.001, Fig. 3A). The metabolic scaling exponents (*α*) and metabolic levels (*Q*_0_) were one-to-one associated and negatively correlated (Fig. 3A,B). For the IA vegetation, the points were concentrated in the interval of low metabolic scaling exponents and high metabolic levels (the dashed-circle area in Fig. 3B).

**Table 1:** Parameters for regression analyses of leaf mass (*M*_*leaf*_, g) and aboveground biomass (AGB, g).

Plot	n	***M***_***leaf***_ (g)		AGB (g)	
		Mean ± SE	Range	Mean ± SE	Range
40–50 m	42	139.77 ± 20.70	0.24–5570.56	1176.06 ± 178.48	0.27–48005.12
50–60 m	81	4.21 ± 0.08	0.22–33.12	5.42 ± 0.10	0.22–42.24
60–70 m	67	47.59 ± 4.06	0.31–2129.92	251.24 ± 22.85	0.45–11714.56
70–80 m	30	4.27 ± 0.33	0.26–49.60	5.35 ± 0.37	0.32–54.08
80–90 m	18	8.10 ± 0.54	0.31–27.84	10.39 ± 0.71	0.43–39.96
NIA	65	3.42 ± 0.11	0.10–96.00	4.76 ± 0.19	0.13–125.04

### Community structures and ecosystem functions

**Figure 4:**
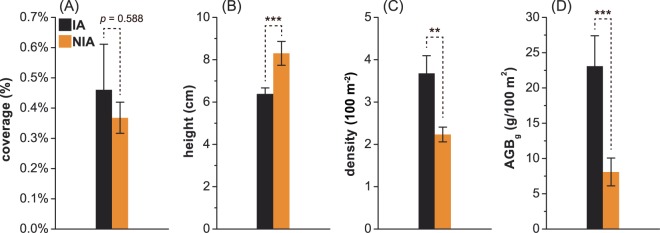
The differences in the community structures and ecosystem functions between interfering areas (IAs) and non-interfering areas (NIAs). (**A**) to (**D**) represent the spatial averages of coverage, height, density, and aboveground biomass of grass (AGB_g_), respectively. The error bars indicate standard errors. “*”, “**” and “***” indicate the significance levels of 0.05, 0.01 and 0.005, respectively.

**Figure 5:**
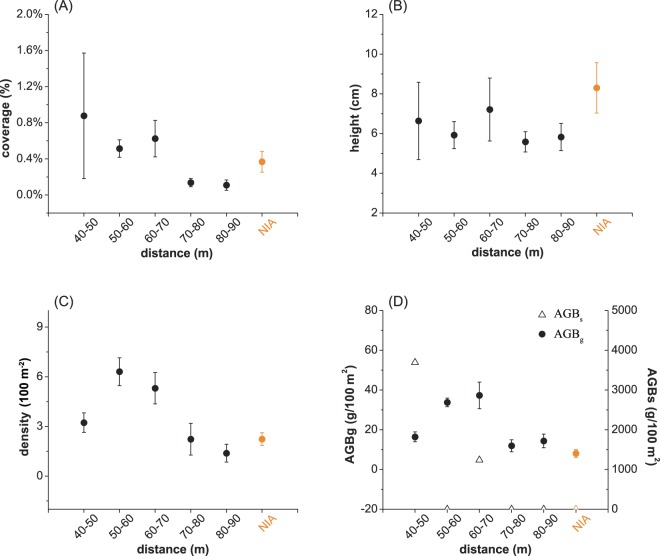
The changes in the community structures and ecosystem functions at various distances from the wind turbines. Changes in coverage (**A**), height (**B**), density (**C**), and AGB (**D**). Solid circles and empty triangles represent the aboveground biomass of shrubs (AGB_s_) and grass (AGB_g_), respectively. Orange and black represent the non-interfering area (NIA) and interfering area (IA) plots, respectively. The error bars indicate standard errors.

For community structures, significant differences were found between the IA and NIA communities (Fig. 4). The coverage of IA vegetation was higher, but not significantly, than that of NIA vegetation (*P* = 0.588, Fig. 4A). The community coverage at the distances of 50–60 m (mean ± standard error, 0.51 ± 0.098%) and 60–70 m (0.63 ± 0.20%) were significantly higher than those at the distances of 70–80 m (0.14 ± 0.046%), 80–90 m (0.11 ± 0.059%) and those of the NIA communities (0.37 ± 0.15%), and the differences were significant (KW post hoc: *P* < 0.005; Fig. 5A). The community coverage at the distance of 40–50 m presented a large variance (0.88 ± 0.69%) and was not significantly different from the groups at other distances (KW post hoc: *P* > 0.05). In contrast, the height of the NIA communities (8.30 ± 1.27 cm) was significantly higher than that of the IA communities (*P* < 0.005, Fig. 4B). The density and AGB were both significantly higher in IAs than in NIAs (*P* < 0.01, Fig. 4C,D). The highest density occurred at 50–60 m (6.31 ± 0.84 100 m^−2^) and 60–70 m (5.31 ± 0.94 100 m^−2^), and the densest plots (50–60 m) were nearly three times as dense as those in NIAs (2.23 ± 0.96 100 m^−2^, Fig. 5C). Because the emergence of large shrubs will cause a significant increase in AGB, we analysed the AGB of shrubs (AGB_s_) and grasses (AGB_g_) separately. Similar to the distribution density, the highest AGB_g_ occurred at 60–70 m (37.29 ± 6.70 g), followed by 50–60 m (33.76 ± 2.16 g), where values were over four times that of the NIA classes (8.09 ± 1.98 g, Fig. 5D). No shrubs were found in the NIA communities; however, shrubs appeared in the IA communities in the 40–50 m and 60–70 m plots, and the AGB_s_ values reached 3692.70 g and 1238.52 g, respectively, which were well above the values of the AGB_g_, which ranged from 11.95 g to 33.76 g (KW post hoc: *P* < 0.005; Fig. 5D).

The results showed that the height of the community was positively correlated with the wind velocity, while the density was negatively correlated with the wind velocity, both at the 0.05 significance level (Table 2). Hence, changes in the wind velocity caused by wind turbines had opposite effects on the height and density, respectively. Wind velocity was not significantly correlated with other functional traits, including the ratio of leaf to stem, leaf water content, stem water content, coverage, and AGB (Table 2). Six paired correlations appeared to be significantly different (underlined values in Table 3). Much higher correlation coefficients were identified for stem water content and leaf water content, height and stem water content, and coverage and height in interfering conditions (Table 3).

**Table 2:** Correlation analysis among wind velocity and related vegetation traits at different scales.

Item	LvsS	LWC	SWC	H	D	CVR	AGB
Wind velocity	−0.087	0.045	0.032	0.255*	−0.214*	−0.030	−0.072

Including: ratios of leaf biomass and stem biomass (LvsS), leaf water content (LWC), stem water content (SWC), height (H), density (D), coverage (CVR) and aboveground biomass (AGB). Here, “*” and “**” indicate the significance levels of 0.05 and 0.01, respectively.

**Table 3:** Correlation analysis and comparison of vegetation traits within interfering area and non-interfering area plots.

Item		LvsS	LWC	SWC	H	D	CVR	AGB
LvsS	IA	1						
	NIA							
LWC	IA	0.222	1					
	NIA	−0.066						
SWC	IA	0.329*	0.582**	1				
	NIA.	0.272	0.198					
H	IA	−0.213	−0.229	−0.473**	1			
	NIA	−0.243	0.257	−0.269				
D	IA	−0.101	−0.070	0.108	−0.136	1		
	NIA	−0.048	0.175	0.017	−0.238			
CVR	IA	−0.148	−0.193	−0.405**	0.855**	0.180	1	
	NIA	−0.288	0.143	−0.416*	0.370	0.557**		
AGB	IA	−0.044	0.145	−0.023	0.178	0.448**	0.263*	1
	NIA	−0.275	0.039	−0.480*	0.482*	0.340	0.799**	

Including: ratios of leaf biomass and stem biomass (LvsS), leaf water content (LWC), stem water content (SWC), height (H), density (D), coverage (CVR), and aboveground biomass (AGB). IA and NIA indicate interfering area and non-interfering area plots, respectively. Here, “*” and “**” indicate the significance levels of 0.05 and 0.01, respectively.

## Discussion

This study demonstrated that inland wind farms in deserts had positive ecological effects on vegetation during operation, which was contrary to the conclusions in the literature showing that the presence and operation of wind turbines had negative environmental impacts^3^. The positive effects can be explained at three different scales. At the individual scale, plants experienced a better growth environment and were in a better physiological state under the protective conditions provided by wind turbines. Considering the same or similar species, the metabolic scaling exponent (*α*) of individuals varies at different physiological states^34^. The value of *α* is noticeably larger (>0.75, or even >1) when water is severely limited^41^ or in the case of less light and low temperatures^42–45^. In this study, the significantly lower *α* in the IAs indicated that wind turbines mitigate drought stress, thus promoting the metabolism of plant individuals (indicated by Q_0_).

At the scale of community structure, the IA plants grew shorter but at a higher density (Fig. 5B,C), and the promotion of lateral growth over vertical growth resulted in a higher coverage condition than that of the NIA plants (Fig. 5A and Table 3). The difference in coverage was not significant, which may be due to the large variance in the coverage between 40 m and 50 m within the IA (Fig. 4C). Meanwhile, the significant positive correlations between coverage and density and between coverage and biomass indicated that the communities under the influence of wind farms had a stronger emerging trend than those in the NIA regions, where the communities were scattered (Table 3). In this study, the changes in plant communities were significantly correlated with the wind variation. The results were in accordance with the previous conclusions that wind farms could change the local microclimate and have a noticeable impact on the local vegetation and ecosystem^46^. However, how wind wake affects precipitation, evapotranspiration or soil moisture and further vegetation remains unclear. For example, the postulated effect of wind turbines on evapotranspiration showed an increasing trend of 0.2 mm h^−1^ during stable operation^12^; however, there were no field data supporting this hypothesis. Therefore, the quantitative relationship between local climate change and plant variation and the corresponding response of vegetation to this change have yet to be explored. Moreover, the turbine-generated climate change and the natural climate inter-annual variability should be separated during an assessment^47^.

**Figure 6:**
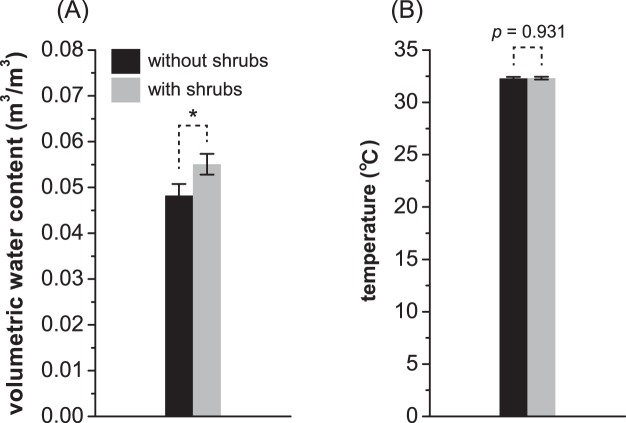
Comparison of the volumetric water content (**A**) and temperature (**B**) of soils with and without shrubs in interfering areas (IAs). “*” indicates a significance level of 0.05. Error bars indicate standard errors. Grey and black represent the soil conditions with and without shrubs, respectively.

Under the influence of wind farms, shrub species emerged, and overall AGB increased. The increase in biodiversity showed that the functions of the Gobi ecosystem had been greatly improved, as more diverse systems are expected to be more stable against perturbations and extreme events^48–50^. Shrubs are important modulators of physical conditions, including water availability, microclimates and soil nutrients, which are crucial for other communities in arid ecosystems^51–54^. In deserts, shrubs around wind turbines may improve microclimate (moister, Fig. 6) by shading to reduce evapotranspiration and reducing wind speed, thus providing a more suitable habitat for shrubs. It is convenient with previous studies that grasses emerging below shrubs may have a higher survival rate and a better physiological status than those that germinated in the open areas between shrubs^55–57^.

The influences of wind farms on ecosystems are inevitably dependent on species and location^46^. In this study, the Gobi Desert is characterized by distinct simple structures, poor productivity and extremely harsh environmental conditions, which are distinct from the characteristics of coastal ecosystems. The desert ecosystem is highly vulnerable to any unfavourable disturbances due to a lack of water and is sensitive to environmental changes^24^. Using climate models, Li *et al*.^58^ found that large-scale wind farms in the Sahara caused a two-fold increase in precipitation, and the resulting increase in vegetation, led to enhanced precipitation. Our results support these simulated results, suggesting that large-scale wind farms in deserts have a positive effect on vegetation by improving microclimate. Therefore, compared with other systems with small-scale, scattered distributions, and low precipitation-vegetation feedback, changes in environmental conditions resulting from wind turbines could have a much greater effect on desert vegetation, which could be the main reason for the positive ecological effects of the Gobi wind farms. In other studies, similar positive effects were also found for specific systems and species. For instance, some land animals (e.g., tortoise) benefit from the enhanced food availability, low traffic and declining predator populations in the new habitats provided by wind power facilities^59,60^. In some benthic environments, new communities are established, and local fish richness increases because the wind turbine foundations may provide highly heterogeneous rocky-to-sandy habitats over the original homogeneous sandy habitats^61–64^. Thus, future research should quantify the effect of local habitat changes on different species so that the construction mode of wind power can be improved by reinforcing positive effects and avoiding negative effects.

Another explanation for the positive effect of wind farms observed in this study may be the strong edge effect of the areas of cleared ground, as well as that of the roads and transmission lines of wind power facilities (Fig. 1B). Surface run-off from precipitation collected along desert roads provides more water for vegetation along the road curbs than for vegetation located away from the road, resulting in enhanced productivity, diversity and perhaps even stability^65,66^. Port and Thompson^67^ found that roadside plants benefited from passing cars because they acquired additional nitrogen from exhausted gas. Plants in the vicinity of the desert transmission lines were also found to exhibit similar growth trends, i.e., they were larger, more vigorous and productive. Bolling and Walker^68^ reported that the decompaction of roads and the amelioration of micro-topography heterogeneity will increase the probability of a more natural community succession and lead to a smooth development trajectory. Although the disturbance of these facilities was avoided as much as possible in our study, the effect of the area of cleared ground within the 40 m radius could not be completely ignored, as these areas were adjacent to the 40–50 m quadrats. This adjacency could be responsible for the large variance in vegetation coverage in these areas closest to the area of cleared ground (Fig. 5A) and contribute to the non-significant difference between IAs and NIAs (Fig. 4A). It should be emphasized that this situation is artificial, which is away from a natural development of the plants in their habitats. However, the potential edge effects of wind turbines are worthy of our attention and should be used in the future design of wind farms to maximize their ecological benefits.

This study is the first to report the effects of wind farms on vegetation at different scales using site experiments. It is true that all facilities used for renewable energy production occupy a large area of land and require roads and transmission lines, thus resulting in habitat loss or change during the facility’s construction phase^4,69,70^. However, for wind energy, the ecological effects were smaller than those of other renewable energies^70^, and offsetting schemes could be implemented during the operation phase. The results further indicated that building wind farms in the Gobi (i.e., a good site selection) can utilize abundant wind resources with low ecological effects. Although the current wind development route of China is from inland to coastal and offshore farms^20^, we found empirical ecological evidence for this win-win development mode for both resources and ecology in an extremely harsh environment. Furthermore, based on our results, shrubs, such as *Nitraria sphaerocarpa* Maxim. and *Ephedra przewalskii* Stapf, can be artificially planted to improve the environment around the wind turbines during the operation phase.

## Conclusions

Understanding the ecological effects of large-scale wind farms in Gobi ecosystems is one of the key objectives of sustainable development. To our knowledge, this study is the first to report the effects of Gobi Desert wind farms on vegetation. This study indicates that wind farms have positive ecological effects on vegetation in desert ecosystems at different scales. Under the influence of wind turbines, plants were more metabolically efficient, with higher community coverage, density, and AGB. A strong correlation was found between the changes in the community structure and the local climatic variation caused by wind turbines. Thus, building wind farms in desert areas that utilize wind energy is a cleaner production method that simultaneously promotes the development of local vegetation. It is a win-win strategy that both contributed to the growth of desert vegetation with a favourable microclimate and sufficiently utilized wind power to produce clean energy. In the future, long-term studies are required to explore the mechanism of how vegetation changes in response to variations in environmental factors caused by facilities. In practice, the positive effects of wind turbines could be better utilized in the ecological compensation and restoration of the Gobi Desert. Evaluating the impacts of habitat changes caused by wind power facilities on different systems and different animals requires a comprehensive assessment, as it is important for energy policy making. In addition to wind power in the Gobi Desert, studies should further explore whether other renewable energy facilities have similar ecological effects in deserts and other extreme environments.
